# The Piriform Cortex and Human Focal Epilepsy

**DOI:** 10.3389/fneur.2014.00259

**Published:** 2014-12-08

**Authors:** David N. Vaughan, Graeme D. Jackson

**Affiliations:** ^1^Florey Institute of Neuroscience and Mental Health, Heidelberg, VIC, Australia; ^2^Department of Neurology, Austin Health, Heidelberg, VIC, Australia; ^3^Department of Medicine, University of Melbourne, Melbourne, VIC, Australia

**Keywords:** pyriform, area tempestas, claustrum, olfaction, olfactory aura, EEG-fMRI, temporal lobe epilepsy, intracranial electrodes

## Abstract

It is surprising that the piriform cortex, when compared to the hippocampus, has been given relatively little significance in human epilepsy. Like the hippocampus, it has a phylogenetically preserved three-layered cortex that is vulnerable to excitotoxic injury, has broad connections to both limbic and cortical areas, and is highly epileptogenic – being critical to the kindling process. The well-known phenomenon of early olfactory auras in temporal lobe epilepsy highlights its clinical relevance in human beings. Perhaps because it is anatomically indistinct and difficult to approach surgically, as it clasps the middle cerebral artery, it has, until now, been understandably neglected. In this review, we emphasize how its unique anatomical and functional properties, as primary olfactory cortex, predispose it to involvement in focal epilepsy. From recent convergent findings in human neuroimaging, clinical epileptology, and experimental animal models, we make the case that the piriform cortex is likely to play a facilitating and amplifying role in human focal epileptogenesis, and may influence progression to epileptic intractability.

## Introduction

One of the important human senses and one of life’s great pleasures is olfaction. From the aroma of a floral bouquet, to the flavor of a meal, and even to the familiar scent of a family member, odors provide us with rich information about our environment that influences our decisions, emotions, and memories.

The piriform cortex is a unique brain region that underlies the mechanisms that produce these olfactory experiences. It forms the major part of the primary olfactory cortex and has extensive connections with other parts of the olfactory network. It is a phylogenetically old structure that can also be found in amphibians, reptiles, and other mammals, and as such has a number of special properties. Unlike other primary cortical regions, it receives input directly from the olfactory bulb without this information being relayed through the thalamus. Additionally, it has a three-layered allocortical structure, which in human beings is otherwise only found in the hippocampus – one of the regions most implicated in focal epilepsy.

Historically, the role that the piriform cortex may play in epilepsy has not been widely recognized. In the study of human focal epilepsy, attention has mostly been given to mesial temporal structures, especially the hippocampus, and to regions of abnormal brain structure. The earliest indications that seizures may involve olfactory cortex were descriptions in the late nineteenth century of “uncinate seizures,” which begin with an olfactory hallucination, and were generally thought to herald a progressive tumor of the temporal lobe. Separately, the clinical observation that some people with epilepsy have impaired olfactory function also hinted at seizure involvement of olfactory cortex. It was not until the 1980s that the particular epileptogenicity of the piriform cortex in animal models was discovered, although this finding did not have an immediate impact on human clinical epileptology.

Over the last two decades, the application of functional neuroimaging to human brain function has led to many new insights into the role of the piriform cortex in olfactory perception. In the field of epilepsy, similar techniques have emphasized a network view of seizures. Most recently, several studies using data from electroencephalography, functional MRI and nuclear medicine imaging, have suggested that the human piriform cortex may be a common node in focal epilepsy arising from different brain regions.

Therefore, it is now timely to revisit the piriform cortex and to re-examine its relevance to focal epilepsy. Beginning with a description of the anatomy and function of the piriform cortex, we go on to review the literature regarding seizures that arise within olfactory cortex in animal models and human beings, the involvement of piriform cortex in distant inter-ictal discharges, and the impact of epilepsy on olfaction. Finally, we discuss the potential for the piriform cortex to become a therapeutic target in treatment of epilepsy, and describe a case of possible piriform epilepsy where resection of the piriform cortex was performed. From consideration of these convergent lines of evidence, we argue that the piriform cortex is critically placed between limbic and cortical networks, to distribute epileptic activity, facilitate epileptogenesis, and potentially contribute toward the development of intractable human epilepsy.

## The Anatomy and Function of the Piriform Cortex has Properties That Predispose it to Epileptic Seizures

Synonymously referred to as “piriform,” “pyriform,” and sometimes “prepyriform” (indicating the anterior piriform), the piriform cortex is the largest component of primary olfactory cortex (1–3).

Odors are first detected in the nasal epithelium by olfactory sensory neurons. These cells project to the olfactory bulb, where inputs from similar receptor types are collected together in glomeruli. Here, they synapse onto mitral and tufted cells, which project to cortical regions via the olfactory tract (4).

Primary olfactory cortex is defined as regions that receive direct input from the lateral olfactory tract. In addition to piriform cortex this includes the anterior olfactory nucleus, olfactory tubercle, periamygdaloid cortex, and the anterior part of the entorhinal cortex (5). Beyond these regions, the olfactory network includes orbitofrontal cortex, thalamus, and insula cortex (6) and interactions with other cortical networks.

### Anatomical location of the piriform cortex

**Figure 1:**
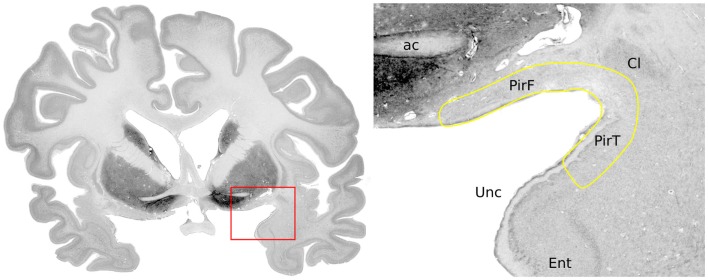
**Anatomical location of the piriform cortex**. Nissl stained coronal brain slice at the level of the anterior commissure of a 65-year-old woman, from the BigBrain dataset (9). Labels were placed with reference to Mai et al. (7): ac, anterior commissure; PirF, frontal piriform cortex; PirT, temporal piriform cortex; Cl, claustrum; Unc, uncus; Ent, entorhinal cortex.

**Figure 2:**
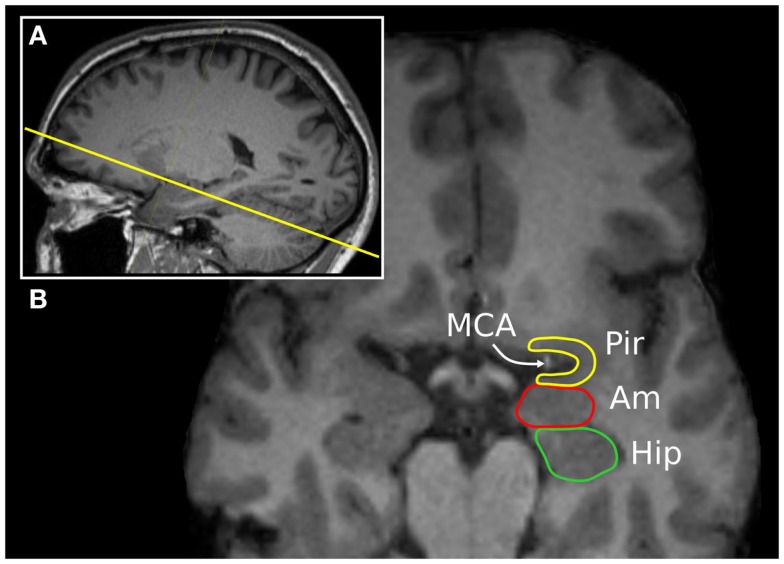
**The “piriform axis”**. T1-weighted MPRAGE image of a 37-year-old man, displayed in a **(A)** para-sagittal and **(B)** oblique-axial orientation, approximately +20° relative to the anterior commissure-posterior commissure axis. This orientation allows the relationship between the piriform cortex (Pir), amygdala (Am), and hippocampus (Hip) to be seen. The arrow indicates the position of the middle cerebral artery within the endorhinal sulcus.

The human piriform cortex is located at the junction of the temporal and frontal lobes, medial to the temporal stem (7), and lines the superior and inferior banks of the endorhinal sulcus (Figures 1 and 2). The name piriform comes from its “pear-shaped” appearance in some mammals such as cats (3), although in human beings, it is a relatively smaller structure and does not have this shape (8).

In human beings, it can be subdivided anatomically into frontal or temporal lobe parts. In the temporal lobe it begins anteriorly at the level of the limen insulae, and extends posteriorly to overlie the amygdaloid nuclei (10), becoming contiguous with the cortical amygdala. Medially, the piriform cortex transitions into the perirhinal or entorhinal cortex, with this border marked more posteriorly by a small depression, the sulcus semiannularis. In the frontal lobe, the piriform cortex extends from the fundus of the endorhinal sulcus, forming a triangular region that is bounded medially by the olfactory tubercle and lateral olfactory tract (11, 12). Laterally, it merges into the insular neocortex (7).

In rats, the piriform cortex is comparatively much larger, and does not have the curvature around a deep sulcus that is seen in human beings. It lies along a rostrocaudal axis, and can be divided into an anterior and posterior part on the basis of the thickness of cell layer III, and the presence of the overlying lateral olfactory tract (3).

Histological studies in the macaque (5) indicate that the primate frontal and temporal piriform cortex correspond to the rodent anterior and posterior piriform, respectively. Despite this, some human MRI studies have divided the piriform cortex into anterior and posterior parts, using at a given *y*-axis value in the MNI coordinate system (13), or at the most anterior coronal slice where frontal and temporal lobes meet (14). Therefore, the specific criteria used in each study to subdivide the piriform should be carefully noted when comparing results.

### Histology of the piriform cortex

The defining histological feature of piriform cortex is its allocortical three-layered structure (11).

The main excitatory neuron types are superficial pyramidal cells, deep pyramidal cells, and semilunar cells. The pyramidal cells are found densely packed in layer IIb and more sparsely in layer III, with dendrites projecting up to layer I to receive inputs from the olfactory bulb (15, 16). Semilunar cells are a distinct population found in layer IIa, which also receive olfactory bulb inputs, and are similar to pyramidal cells but do not have basal dendrites and show a distinct firing pattern (15, 17).

The interneurons of the piriform cortex are mostly inhibitory GABAergic cells. They are found across all layers and multiple classes can be identified on the basis of unique electrophysiological and morphological properties (18, 19). They variously provide both feed-forward and feedback inhibition onto the pyramidal cells (20–23), which allows the pyramidal cells to produce temporally sparse but accurate responses to trains of olfactory bulb input.

The endopiriform nucleus is a separate population of neurons that lies deep to the piriform cortex (24), being found along its full rostrocaudal extent. These multipolar cells project widely to piriform cortex, orbitofrontal, and thalamic regions (25). In rats, the endopiriform nucleus provides a layer of integration between olfactory and gustatory processing (26). The endopiriform nucleus is also found in primates (5). In human beings, it corresponds to the parts of the ventral claustrum that lie adjacent to the piriform cortex and amygdala (27), which have been labeled “prepiriform claustrum” and “periamygdalar claustrum,” respectively (7). These areas should not be confused with the dorsal (or insular) part of the claustrum (28), which has a different embryological origin and different patterns of connectivity (29).

There are several striking similarities between the structure of piriform cortex and that of the hippocampus (8). As paleocortex, both have a phylogenetically conserved structure with three layers, pyramidal neurons with similar morphology, a predominantly horizontal arrangement of fiber projections, and the presence of GABAergic interneurons. Analogous microcircuits in both piriform cortex and hippocampus provide excitation, feed-forward inhibition, and feedback inhibition (30). Their main structural difference is that the piriform does not have a distinct zone that corresponds to the dentate gyrus, although the distributed semilunar cells do have morphology that is similar to granule cells.

### Structural connectivity of the piriform cortex

The main input to the piriform cortex is from mitral cells, and to a lesser extent tufted cells, of the olfactory bulb (15). Each glomerulus in the olfactory bulb, which represents a specific olfactory chemoreceptor type, projects to a broad region of the piriform cortex to synapse with many pyramidal cells (31, 32). Each pyramidal cell receives input from a random selection of glomeruli, allowing cells to respond to complex features of odor mixtures (33).

Additional inputs to the piriform are from the anterior olfactory nucleus and association fibers from all other olfactory cortical regions, as well as lighter commissural projections from the contralateral piriform cortex (34). Neuromodulatory inputs include cholinergic modulation from the horizontal limb of the diagonal band, serotonergic modulation from the raphe nuclei (activating inhibitory GABAergic interneurons), noradrenergic input from the locus coeruleus (35), and dopaminergic modulation from the ventral tegmental area (3, 6).

Within the piriform cortex, pyramidal cells are strongly interconnected, by recurrent projections onto many other pyramidal cells (1). A single pyramidal cell has an arbor that extends over much of the piriform cortex, and in the rat, synapses with more than 1000 other cells (36). This forms a large excitatory network that requires strong local feedback inhibition to prevent runaway activation (20). However, the benefit of this arrangement of diffusely projecting inputs, combined with extensive intra-piriform connectivity, is the ability to perform pattern matching in an architecture described as “content-addressable memory” (37). This allows partially degraded patterns of input to produce consistent reproducible responses that are spatially distributed across the piriform cortex (38).

The outputs from piriform cortex pyramidal cells are widespread to cortical and subcortical regions (15, 39). There are strong limbic connections, especially to the entorhinal cortex and to the amygdala (36, 40, 41), frontal lobe connections to multiple parts of the orbitofrontal cortex, and projections to agranular insular cortex (5). Important subcortical connections are to the mediodorsal nucleus of the thalamus (42, 43), and to the hypothalamus (44). There are also return projections from the piriform to the ipsilateral olfactory bulb, which has been likened to the cortico-thalamic circuit in other sensory modalities by some authors (45).

Based on these connections, several local recurrent circuits may provide a substrate for seizure activity (3). Firstly, the projections from piriform pyramidal cells to amygdala nuclei are returned by projections from the basolateral amygdala to the endopiriform nucleus. Secondly, projections to the subiculum link the piriform with the hippocampus, a loop that is returned to the piriform via the entorhinal cortex. Finally, piriform feedback to the olfactory bulb could also form a reentrant circuit (46).

### Functional role of the piriform cortex within olfactory networks

The perception of odors involves activation of distributed cortical and subcortical networks, with regional nodes that are variably recruited depending on the nature and complexity of the olfactory task (47). Activation of the piriform cortex is seen commonly across all olfactory tasks, and it appears to be the key region for representation of the “olfactory object” (48). However, the piriform also has an important role in discrimination of odors (49), in olfactory working memory (50), and acts as an information distributing node to other brain regions (51).

Within piriform cortex, odors are represented as spatially distributed ensembles (52, 53). This activity is not static over time, and shows variability with the phase of the respiratory cycle (54), and especially with sniffing (55). The anterior piriform cortex encodes for molecular features of the odorant, whereas the posterior piriform encodes for the quality of the odor (14). There is rapid habituation of the piriform response to a sustained odor within seconds (56), which is a property that may be the basis for figure-ground segmentation, that is, to allow a novel odor to stand out in a complex olfactory environment (48). Piriform activation can also occur in the absence of an odorant, for example, by imagining a smell (57), or on viewing a picture or word that has a strong olfactory association (58, 59), which is consistent with the behavior of primary cortical regions for other sensory modalities.

Larger scale network interactions of the piriform cortex can be conceptualized as including an orbitofrontal-thalamic circuit, a limbic stream, and a fronto-temporal cortical stream. Additional cortical regions including the anterior insula are important for integration of olfaction into taste and flavor (60).

The orbitofrontal cortex is the principal higher-order target for piriform cortex, both directly and indirectly via the thalamus. The olfactory functions of orbitofrontal cortex include involvement in encoding for odor identity and valence, predicting anticipated olfactory stimuli (61), multisensory integration of olfactory information, assessment of reward and value signals, and a role in emotion (62). The mediodorsal nucleus of the thalamus provides an indirect pathway between piriform cortex and the orbitofrontal cortex, and is therefore well placed to provide assessment of prediction error (63), or to control olfactory attention (64). Furthermore, the connectivity between these three regions is modulated during olfactory learning (65), and by olfactory attention (66).

Limbic processing of olfactory stimuli plays an important role in memory, emotion and social behavior. Indeed the spontaneous recall of a vivid memory or emotion on smelling a particular odor is a common human experience (67). Entorhinal cortex and hippocampal activation occurs during odor identification and memory tasks, reflecting the involvement of autobiographical memory systems (68). Exposure to odors of varying degrees of pleasantness produces amygdala activation that reflects the valence (69) and also the intensity and overall emotional value of an odor (70).

The semantic network, which involves the dominant inferior frontal gyrus and its downstream influence on the fusiform gyrus and posterior temporal regions, is important for naming odors and for olfactory working memory when odors are nameable (50, 71). The temporal pole may be the critical area for interaction between olfactory and semantic networks based on an apparent disconnection syndrome in people with atrophy of this region (72).

## Piriform Cortex is the Most Susceptible Region to Epileptogenic Stimulation

### Piriform cortex sensitivity to chemical stimulation

A unique property of the piriform cortex is its sensitivity for inducing epileptic seizures in experimental animals. In 1985, Piredda and Gale identified a site in the forebrain of the rat, which is exquisitely responsive to pro-convulsant chemical stimulation (73), naming it the “area tempestas” (74). Injections into this region produced bilateral clonic seizures, at much lower concentrations than are required when applied to other brain regions. Picomolar amounts of bicuculline (a GABA antagonist), carbachol (a cholinergic agonist), kainic acid (an excitatory amino acid), and micromolar concentrations of glutamate all demonstrated this effect. Preventing glutamatergic excitation via either AMPA or NMDA receptors in the area tempestas can prevent seizures, indicating that both receptor types are needed for this regions to become epileptogenic (75, 76).

The location of the area tempestas is deep to the anterior piriform cortex, overlapping cellular layer III and the adjacent endopiriform nucleus (25, 73). Some studies have shown wider sensitivity to bicuculline across both anterior and posterior piriform cortex, however, and some variability in the expression of seizures between different rat strains (77).

The area tempestas cannot be ethically demonstrated in human beings, as it is defined by an epileptic response to chemical stimulation, and is not a circumscribed anatomical structure. This study has been performed in non-human primates, however (78), using bicuculline injections into the frontal piriform cortex. A highly focal 2 mm region of chemosensitivity was identified. The resulting seizures consisted of automatisms and myoclonus of the mouth and face, contralateral arm clonus, salivation, behavioral arrest, and unresponsiveness, with retained postural control (79), consistent with the features of focal dycognitive and focal motor seizures in human beings.

The brain regions most affected by seizure activity triggered from area tempestas, are the posterior piriform cortex and ipsilateral entorhinal cortex (80), the olfactory bulbs, perirhinal cortex, amygdala, and the mediodorsal thalamus (81). This has been demonstrated by ictal uptake of radiolabeled glucose, and also by the ictal expression of *c-fos* and other immediate-early genes *in vivo* (82–84). Examination of *in vitro* slice preparations shows that discharge propagation from the endopiriform nucleus up to the superficial layers of piriform cortex is via longitudinally orientated rostrocaudal association fibers (85).

The posterior piriform cortex, perirhinal cortex, and mediodorsal thalamus are important regions for seizure propagation from the area tempestas. Blockade of glutamatergic transmission at these locations prevents such seizures occurring (76, 80, 81). This is mediated primarily by the action of AMPA receptors, as selective blockade of NMDA receptors did not prevent seizures occurring.

### Piriform susceptibility to electrical kindling

Seizures may also be produced from the piriform cortex by repeated electrical stimulation (3, 86). Comparison to other nearby structures shows that perirhinal cortex and dorsal claustrum also kindle as rapidly, or even faster (87). The amygdala, entorhinal cortex, and hippocampus are less sensitive (88).

The location within piriform cortex for the most rapid kindling in rodents has been reported as the central part (layer III of the rostral part of the posterior piriform cortex) (89) or in the endopiriform nucleus (90). Deep layers of the posterior piriform cortex also show the lowest afterdischarge thresholds. In human beings, these areas correspond to the frontal piriform close to the temporal stem, or to the prepiriform claustrum. Several authors have emphasized that the region corresponding to area tempestas (in the deep anterior piriform cortex) does not respond to electrical kindling as quickly (91, 92).

Seizures produced during the course of piriform kindling follow the same progression of motor features as kindling in other limbic regions (93). During piriform-kindled status epilepticus (type 2), where the animals show intermittent freezing and exploratory behaviors, the affected regions are the olfactory cortex and amygdala. When facial and limb clonus was also present (type 3), the hippocampus, prefrontal cortex, and insular cortex were also seen to be involved (94). Piriform kindling produces chronic network-wide changes, for example, altered potentiation at the entorhinal cortex (95), which may relate to emergence of spontaneous seizure after kindling is completed.

Therefore, the piriform cortex is highly susceptible to the induction of seizures by both chemical and electrical means, although the exact positioning of the intervention within the piriform appears to be less important, than whether an extended olfactory-limbic network can be recruited.

## Human Seizures with Olfactory Auras Tell us about Epileptic Involvement of the Olfactory Network

Focal seizures that begin with an olfactory sensation as their earliest feature can be inferred to arise within the olfactory network. In human beings, this is a relatively uncommon type of seizure, but examining these events in detail can tells us about the patterns seizure spread from the piriform cortex. The most common olfactory ictal phenomenon is a hallucination, where the perception of an odor is unrelated to any environmental stimulus. There can also be olfactory illusions, where odors in the environment are misperceived (96, 97), or vaguer episodes with the quality of a reminiscence (98).

The earliest influential descriptions of seizures with an olfactory aura were by Hughlings Jackson in 1889. He described a woman who developed stereotyped episodes of a horrible smell of “dirty burning stuff” associated with a complicated visual hallucination and a feeling of suffocation. A sarcoma of the “temporo-sphenoidal lobe” was found at postmortem. Review of their diagrams shows invasion of the piriform cortex, temporal pole, amygdala, adjacent white matter, and compression of the lenticular nucleus (99). Subsequently the name “uncinate group of fits” was given to seizures beginning with a crude sensation of smell or taste, and variably associated with oral automatisms and the “dreamy state” (100, 101). Importantly, this label was intended to convey that these seizures involved a broad region of which the uncus is a part, and should not be interpreted as a precise anatomical localization.

### Clinical characteristics of seizures with an olfactory aura

Estimates of the prevalence of olfactory epileptic auras are quite variable due to patient selection criteria and how auras were ascertained. Considering all people with focal epilepsy, rates between 0.9 and 8.1% are reported (102–107). If restricted to epilepsy arising from the temporal lobe, with or without selection for epilepsy surgery, olfactory auras are present in 0.6–16% (108–113). Out of people who experience an epileptic aura of any kind, between 19 and 30% have an olfactory aura (114–116).

The character of olfactory hallucinations is usually unpleasant, and may be described as rotten, fetid, sulfurous, or burned (110, 117). This may correspond to epileptic activity causing particularly intense activation of the piriform cortex and amygdala, as occurs with non-pathological smelling of unpleasant odors in the environment (57). Less commonly the olfactory hallucination is neutral and only rarely pleasant (102, 106). Some descriptions have emphasized the “crude” nature of the experience, without having the full experiential quality of smelling an actual odor (100). Indeed many patients find the hallucination “indescribable,” or refer to it as “like” the aroma of something else (98), suggesting that the engagement of the olfactory network is not the full physiological pattern of olfactory perception. The olfactory hallucination is usually pervasive, but in rare cases is experienced as coming from one nostril (98), or from one side of the body (118), which may be due to lateralized involvement of primary olfactory cortex or activation of the superior temporal gyrus (12).

A particular “rhinostomal” sensation of tickling or pressure in the nose or pharynx often accompanies olfactory hallucinations (98). This may be analogous to the trigeminal nerve stimulation that is physiologically produced by many odorants. A similar sensation of unilateral itching inside the nose has also been triggered by electrical stimulation near the olfactory bulb (119).

Olfactory auras may be accompanied by other aura symptoms, pointing to epileptic activation of multiple sensory or cognitive networks. The association of olfactory auras with ictal emotion (120) suggests epileptic co-involvement of olfactory and limbic networks. Olfactory auras are often accompanied by gustatory or psychic auras when the underlying epileptogenic lesion is a tumor (102, 105, 106). However, patients with mesial temporal sclerosis tended to have epigastic sensations and autonomic phenomena accompanying the olfactory aura (110), indicating different spread patterns depending on etiology.

When there are multiple aura types during the same seizure, the order of progression indicates the direction of epileptic spread. In a small case series, one patient with a neocortical temporal lesion had an olfactory aura followed by a sensory aura. Another similar patient had a concurrent olfactory and psychic aura (116). These examples may represent epileptic spread from the olfactory network into cortical networks and limbic networks respectively. Two further patients with mesial temporal sclerosis were described in this cohort. The first had a concurrent olfactory-abdominal aura, and the second had a progression of autonomic, sensory, and psychic symptoms before the olfactory aura emerged. This latter case is an example where the seizure likely began outside the olfactory network, but it became engaged as the seizure progressed. In patients with mesial temporal sclerosis, imaging data has shown that patients with an olfactory aura are more likely to have an accompanying abnormality of the amygdala (121). This suggests that the amygdala may be a possible gateway for seizure spread from mesial temporal into olfactory networks.

The etiology of seizures with an olfactory aura is commonly found to be a tumor (102, 108, 113) or mesial temporal sclerosis (109, 110, 114), with debate over which of these is more common. Other cases have been caused by intracerebral hemorrhage (122), middle cerebral artery aneurysm (118), arteriovenous malformation, head injury (103, 123), and previous encephalitis (124). In rare cases, there is no obvious cause and no structural abnormality is found on MRI (110). It is the anatomical location of these lesions, rather than the nature of the pathology, that is most relevant to the occurrence of olfactory auras, although the close relationship of the middle cerebral artery to the piriform cortex at the endorhinal sulcus should be noted.

### The post-ictal nose-wipe could be explained by an ictal rhinostomal sensation

A movement of the hand to wipe or rub the nose is often observed in the immediate post-ictal phase following focal seizures. It is most common in seizures from the mesial temporal lobe, occurring in more than half of mesial temporal lobe epilepsy patients having video-EEG prior to surgery (125, 126), but it may also be seen in frontal lobe epilepsy. It may be accompanied by post-ictal coughing (127), and does not occur if the seizure evolves to a bilateral convulsion (119). Typically, the hand ipsilateral to the seizure focus is used, because of contralateral neglect or weakness (125).

We hypothesize that the ictal nose-wipe is a voluntary action performed in response to the ictal rhinostomal sensation, as a result of epileptic activation of olfactory regions. Geyer et al. (119) have previously suggested that olfactory hallucinations and post-ictal nose-rubbing are linked by epileptic involvement arising from the uncus. However, many patients with post-ictal nose-wiping do not have awareness of any olfactory aura (128). Hirsch et al. (125) proposed that the nose-wipe is caused by increased nasal secretions from ictal activation of autonomic pathways, particularly the amygdala (129). Intracranial EEG recordings from the amygdala can show early ictal involvement in seizures, which include nose-wiping, but this is neither sufficient nor necessary for nose-wiping to occur (130).

### Lesion “localization” in seizures with an olfactory hallucination

The anatomical location of epileptogenic lesions indicates how seizure discharges gain access to the olfactory network. It should not be assumed however that the lesion equates to the location where the aura is produced, as emergence of an olfactory percept likely requires coordinated activation of multiple olfactory brain regions (131, 132).

Olfactory auras are not lateralizing, and are associated with similar rates of left and right-sided lesions (107, 110, 111). The most common location is in the anteromesial temporal lobe, with some tumors extending into the frontal lobe (102, 108). Other series have found only temporal lobe lesions, both with and without involvement of mesial temporal structures (107, 114). In a few cases, lesions have been isolated to the amygdala (102, 110, 123).

The case most strongly indicating primary involvement of the piriform cortex is provided by Mizobuchi et al. (118). The cause of seizures was a 1 cm aneurysm of the middle cerebral artery, “between the tip of the right temporal lobe and the orbitofrontal gyrus.” MRI clearly shows compression of both frontal and temporal piriform cortex, although the authors do not label it as such. The olfactory aura was followed by a phase of retained awareness and speech, but impaired memory, suggesting limited seizure spread to either autobiographical (limbic) or perhaps semantic (cortical) memory networks.

Whether purely frontal lobe lesions can cause seizures with an olfactory hallucination is less clear, even though this is often said to be the case (106, 133). In some series of patients with frontal lobe epilepsy, confirmed by curative frontal lobe resection, there have been no instances of olfactory auras (134). Possible cases include one out of a series of 28 patients with extra-temporal focal epilepsy studied by intracranial stereo-EEG, although this patient was cured by temporal lobe resection (135). Another study described two patients under the heading of an olfactory-gustatory-fear aura, who had frontal lobe lesions at the supplementary motor area and lateral premotor cortex, respectively (105).

The only unequivocal report of a frontal lobe lesion causing an epileptic olfactory aura was due to an abscess at the frontal pole (136). Several pathways to involvement of the olfactory network are possible here; seizure activity could have spread from the lesion into adjacent orbitofrontal cortex, activity could have propagated via the uncinate fasciculus into the temporal lobe, or there may have been local inflammatory or epileptic irritation of the olfactory tract. Of these sites, an olfactory hallucination has been produced most consistently by electrical stimulation of the olfactory tract (137).

### Ictal EEG in seizures with olfactory auras

Scalp EEG during seizures with an olfactory aura has shown epileptiform discharges at the ipsilateral sphenoidal electrode, consistent with seizure involvement of mesial temporal structures (102, 120, 138). This confirms that these olfactory hallucinations are epileptic in origin, and are not due to mere inter-ictal dysfunction of the olfactory network.

Intracranial EEG recording has much greater sensitivity for detecting focal epileptic activity, but sparse spatial sampling often limits the precision of localization. Electrodes have typically been placed into mesial temporal structures, over the lateral and inferior temporal lobes, and into frontal regions, with the piriform cortex seldom being an explicit target for recording.

The following five reports demonstrate intracranial recordings of epileptic activity in the temporal and/or frontal lobe associated with an olfactory aura, although it must be noted that no cases had an electrode directly in piriform cortex. (i) Epileptic activity at the amygdala and hippocampus was seen in a patient who had a temporo-basal cyst and habitually experienced an epigastric-olfactory-gustatory aura (139). (ii) Discharges from the hippocampus were seen in two patients who had mesial temporal sclerosis, during an olfactory aura (140). (iii) A patient with a more elaborate aura, consisting of initial déjà vu then an olfactory hallucination, detachment, fear, and auditory illusions, had ictal rhythms that were widespread across the right hippocampus, amygdala, anterior cingulate, middle, and superior temporal gyri (141). (iv) A patient who experienced seizures with a sense of foreboding, dissociation, and a “sickening” smell, showed initial activity in superior temporal electrodes, with consistent spread of the discharge into orbital areas (135). (v) A further three patients had simultaneous epileptic activity in the temporal and orbitofrontal regions during the olfactory aura, however, no aura occurred in seizures when only the frontal lobe was involved, or when temporal lobe involvement was late (142).

Therefore, the seizures that produce olfactory hallucinations typically involve relatively widespread activity in the orbitofrontal and anterior temporal lobe. Although recordings directly from piriform cortex were not obtained in these cases, we infer its involvement from its location at the center of the regions that were sampled, and its core role in olfactory perception.

### Intracranial stimulation demonstrates sites that may produce an olfactory hallucination

Direct electrical stimulation of the human brain, either during surgery or via long-term implanted electrodes, has identified locations that may trigger an olfactory sensation similar to the epileptic aura. Findings are somewhat variable, and several large studies of temporal lobe stimulations have induced no olfactory sensations at all (143).

The earliest reports are of a crude sense of smell produced by stimulation of the uncus, or of the olfactory bulb (144). Only a few authors have applied stimulation near the piriform (145). Overall, stimulation of the amygdala is the location that most often produces an olfactory percept, although reproducibility in individual patients is not consistent (145–148).

In one patient with epilepsy, amygdala stimulation produced an afterdischarge that propagated to the hippocampus, at the same time accompanied by a “foul rotten odor” typical of their usual seizures. Transection adjacent to the amygdala prevented propagation to the hippocampus, but the olfactory aura on amygdala stimulation still occurred. The patient became seizure free after resection of the amygdala and overlying anterior cortex, including temporal piriform cortex (123). This suggests that either amygdala activity itself or spread into the adjacent piriform cortex is the relevant pathway, and that amygdala-to-hippocampus spread is less important.

Stimulation over the orbitofrontal cortex does not elicit an olfactory hallucination, unless the electrodes are in a position to stimulate the olfactory bulb or tract (137). The induced odor is always unpleasant. This could be because a large number of fibers are stimulated through the use of macro-electrodes, and the subsequent activation of olfactory cortex, which is relatively intense.

### Seizures may involve the piriform cortex without an olfactory hallucination

An intriguing possibility is that some human seizures may arise from the piriform cortex, without being accompanied by an olfactory aura. This has been demonstrated in a single case of reading epilepsy, that was intensively investigated using combined imaging techniques and advanced statistical modeling (149). Clinically, covert reading induced peri-oral myoclonus, however, no accompanying olfactory hallucination was described. Both magnetoencephalography and EEG-fMRI demonstrated seizure-related activity at the dominant left premotor cortex, with fMRI showing a more extensive network of activation involving left piriform cortex, left thalamus, and right inferior frontal gyrus, consistent with findings from a larger group of people with reading epilepsy (150). Modeling of fMRI timecourses showed the earliest BOLD response was in the left piriform cortex. An effective connectivity analysis identified a model where piriform cortex activity drives activation of the premotor cortex and then onto other regions. While this case report suggests a possible role of piriform cortex in driving seizures into premotor regions, whether this is generally the case in patients with reading epilepsy remains to be confirmed.

## Piriform Cortex is a Node for Secondary Spread of Inter-Ictal Discharges in Human Beings

**Figure 3:**
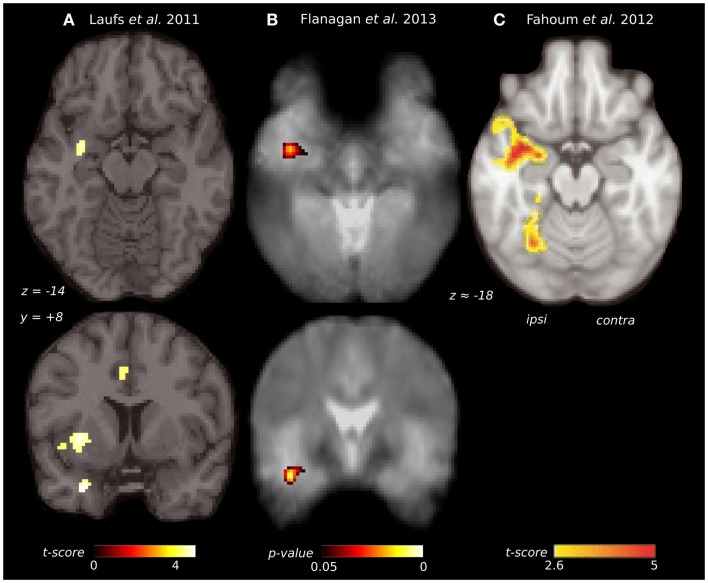
**Comparison of piriform cortex activation in EEG-fMRI studies of focal epilepsy**. **(A)** Group EEG-fMRI analysis for a mixed cohort of focal epilepsy at threshold *p* < 0.001 (*n* = 19). **(B)** Group EEG-fMRI random effects analysis for a mixed epilepsy cohort (*n* = 27) showing *p*-values <0.05 FWE corrected. Reproduced from Flanagan et al. (153) with permission from Elsevier. **(C)** Group EEG-fMRI analysis of a purely TLE cohort (*n* = 32), with hemodynamic response function peaking at 5 s (*p* < 0.05 cluster corrected). Reproduced from Fahoum et al. (154) with permission from Wiley Periodicals, Inc. ©2012 International League against Epilepsy.

Beyond its role in olfactory auras, the piriform cortex may be one of the common pathways for propagation of epileptic discharges in focal epilepsy. The first study to suggest this used combined encephalography and functional MRI (EEG-fMRI) (151). They studied a diverse group of 19 patients, who had focal epilepsy arising from all lobes. After aligning the epileptic side between patients, a second-level random effects analysis was performed, and showed significant clusters with peak BOLD response overlying the ipsilateral piriform cortex (Figure 3A). Regions of activation also extended over the ipsilateral dorsal claustrum and anterior cingulate. The interpretation is that these areas are activated by inter-ictal discharges in many individuals, regardless of the site where the discharge begins. A recent paper has commented that the peak coordinates in this study favor activation of the dorsal claustrum (152), although the shape of the activation clusters do not entirely follow this structure, and activation specifically within the thin sheet of the claustrum would be difficult to resolve at this fMRI resolution.

Replication of this analysis using functional MRI acquired at 3 T, in an independent cohort of 27 patients with heterogeneous epileptic foci, again identified a common area of temporal lobe activation, in the region of the ipsilateral piriform cortex (Figure 3B) (153).

A further EEG-fMRI study analyzed subjects with focal epilepsy, this time grouped by lobe (154, 155). In total, 32 patients had temporal lobe epilepsy, 14 frontal lobe epilepsy, and 20 posterior quadrant epilepsy. The activations detected by this approach were more extensive than in previous studies, suggesting greater homogeneity of epileptic networks within these selected groups. The temporal lobe epilepsy cohort showed an ipsilateral network of activation over the insula, claustrum, temporal piriform cortex, and amygdala (Figure 3C), as well as anterior hippocampus, mid-cingulate and cerebellum. The frontal lobe epilepsy group did not have significant activation of piriform cortex, although other sites of activation were seen in the mid-cingulate, ipsilateral frontal operculum, thalamus, and cerebellum. The posterior quadrant epilepsy group had no significant regions of common activation (154).

Taken together, these results indicate that inter-ictal discharges arising from the temporal lobe, and perhaps those from the frontal lobe, can produce common activation within the piriform cortex, along with other ipsilateral brain regions. This occurs in the absence of positive olfactory symptoms. Whether discharges from parietal and occipital lobe foci also engage the piriform cortex in this way requires further clarification.

## Piriform Cortex is Susceptible to Seizure-Induced Injury and Facilitates Progression of Focal Epileptogenesis

### Status epilepticus injures the human piriform cortex

A characteristic property of the piriform cortex is its tendency to sustain neuronal injury as a consequence of repeated seizures. This is demonstrated by three unusual human cases of status epilepticus, in people who had no prior history of epilepsy (156). The causes were neuroleptic malignant syndrome, carcinomatous meningitis, and unknown, respectively. The duration of status epilepticus on EEG was between 9 h and 3 days. The three individuals died between 11 and 27 days after status epilepticus. At postmortem, neuronal loss was most prominent in the piriform cortex, hippocampal subfields, and amygdala, although with some asymmetry and variability between individuals. Milder widespread changes were seen in the deep layers of the neocortex, the Purkinje cell layer of the cerebellum, and the dorsomedial nucleus of the thalamus. Glutamate-mediated excitotoxicity has been suggested as the mechanism of neuronal necrosis, by analogy to animal studies of status epilepticus.

Domoic acid, a glutamate analog, has also been seen to cause neuronal toxicity in the human piriform cortex following status epilepticus. The most prominent injury is to the hippocampus, which is likely related to kainate receptor excitotoxicity (157), but more widespread injury also occurs, affecting the piriform cortex, olfactory tubercle, amygdala, mediodorsal thalamus, and nucleus accumbens (158). The same pattern is seen with domoic acid in experimental animals (159), although one study in rats has suggested that the most significant early changes are in the olfactory bulb and endopiriform nucleus (160). The mechanism of piriform cortex injury in these cases may be either the direct effect of the toxin, or the kindling effect of repeated seizures.

### Induced status epilepticus injures the piriform cortex in experimental animals

Status epilepticus induced by pilocarpine or kainic acid also produces early injury to the piriform cortex, even though greater attention is often given to the hippocampus in these studies.

Rodents treated with pilocarpine, a potent muscarinic agonist, are often presented as a model of human chronic temporal lobe epilepsy (161, 162). Following systemic administration of pilocarpine, there is an initial phase of limbic status epilepticus, then a latent period of several weeks, before spontaneous recurrent seizures develop. Here, we discuss the initial phase only. Although many brain regions are affected, serial MRI shows the earliest changes in the piriform and entorhinal cortex, as early as 6 h after the status epilepticus (163, 164), reflecting cellular edema, and neuronal loss in these regions (165). Cellular hyperactivity, imaged by *c-fos* expression, is first seen (at 30 min) at the piriform cortex, olfactory tubercle, thalamus, caudate, and lateral habenula, with later changes (at 60–90 min) in hippocampus, amygdala, and basal ganglia (166). Early neuronal loss and gliosis occur in the piriform cortex, hippocampus, amydala, thalamus, and substantia nigra (167). More specifically within the piriform cortex and endopiriform nucleus, it is the posterior two-thirds that are affected, which reflects the pattern of arborization of efferents from the endopiriform nucleus (168). It is primarily the pyramidal cells that are lost, but immunocytochemistry also shows loss of distinct populations of piriform GABAergic interneurons, some of which have analogous labeling to hippocampal basket cells (169). Involvement of the piriform cortex may be explained by cholinergic innervation from the diagnonal band of Broca (170), or the tendency of piriform cortex to produce burst firing with muscarinic antagonism (171). Subsequent neuronal loss may be caused by excitotoxic glutamate release and neuronal calcium influx during seizures (172) or by concurrent ischemic mechanisms (173).

Kainic acid is an analog of glutamate, which like pilocarpine, produces limbic status epilepticus after systemic administration. This results in damage to the hippocampus, amygdala, piriform cortex, entorhinal cortex, thalamus, and septal regions, although with some differences in timing relative to the pilocarpine model (174). The regions showing greatest oxidative stress are the piriform cortex, hippocampus, and cerebellum (175), and the greatest subsequent volume loss is again in the posterior olfactory cortex and amygdala, with loss of approximately one-third loss of neurons in these areas (176). GABAergic neurons of the piriform cortex also show a unique property in this situation, of increasing mRNA expression for glutamate decarboxylase (GAD), perhaps in an attempt to control excitotoxic injury in the face of ongoing neuronal loss (177). The mechanisms of piriform cortex injury here are either the direct excitotoxic effect of the kainic acid, or via release of glutamate during the seizure, and although disentangling these possibilities is difficult, the ability of specific blockade of glutamatergic NMDA receptors to prevent neuronal loss in this model favors the latter (178).

Lesion studies of the piriform cortex further indicate that piriform cortex involvement may be a critical for the development of chemically induced seizures. In the administration of soman, a powerful inhibitor of acetylcholinesterase that causes seizures via stimulation of muscarinic and nicotinic receptors, pre-lesioning of posterior piriform cortex or perirhinal cortex significantly increased the latency to seizure onset. This prevention did not occur with ablation of the amygdala, entorhinal cortex, or hippocampus (179).

### Amygdala kindling causes changes within piriform cortex

Neuronal injury at the piriform cortex, and subsequent change in its function, is seen following electrical kindling at sites such as the amygdala or hippocampus (3). During amygdala kindling, afterdischarges are induced from the piriform from the very first stimulation, indicating the high connectivity from the amygdala and propensity of piriform cortex to sustain epileptic discharges (180). During this process, neuronal loss not only occurs at the primary kindling site, but is also at the central piriform cortex, particularly with loss of GABAergic interneurons (181, 182). Once amygdala kindling is completed, there is increased background firing of neurons in the upper layers of the central piriform. These are most likely inhibitory interneurons, which have pathologically reduced sensitivity to glutamate, and are compensating for loss of feed-forward inhibition (183, 184). There is also increased excitability at the piriform cortex, which is demonstrated by a significant drop in its afterdischarge threshold (185).

Many other changes occur in the piriform cortex following amygdala kindling, which may underlie this increased excitability. These include expression of markers of synaptogenesis on excitatory neurons (186, 187), altered regulation of glutamate transporters (188), abnormal transcription of AMPA and GABA receptor subunits (189, 190), altered expression of voltage gated potassium channels on multipolar inhibitory interneurons (191), alteration of chloride transport that further exacerbates the failure of GABAergic inhibition (192) and proliferation of astrocytes (193). The most recent observation has been the breakdown of perineuronal nets around specific interneurons, leading to increased sites of GABA release, and the pathological rewiring of local microcircuits (194).

### Piriform cortex facilitates epileptogenesis in the amygdala kindling model

As the epileptic state develops, the piriform cortex plays a key role in the facilitation and distribution of kindled afterdischarges. Early in the process, uptake of radiolabeled deoxyglucose during seizures shows involvement only of the amygdala and the regions it is directly connected to, including the piriform. After kindling is completed, much more widespread activation is seen during seizures, affecting substantia nigra, thalamic nuclei, basal ganglia, and bilateral neocortex (3, 195). Similarly using *c-fos* expression as a marker of cellular activity, a limited expression of seizures is seen in the early phases, confined to either a unilateral amygdala-insula-temporal network, or a bilateral amygdala-hippocampal network. Following kindling, this becomes much more extensive involving extensive amygdala, olfactory, hippocampal, and neocortical regions bilaterally (196). Furthermore, during amygdala kindling, spontaneous discharges arise most frequently from the piriform cortex (197). Together this suggests that the piriform is involved in converting the kindled seizure discharge from one that is confined to the stimulation site and immediate projections, into an event having more widespread distribution (3).

The role of the piriform cortex in facilitating epileptogenesis can be further explored by blocking it prior to the kindling process. This approach has given variable results depending on the site and method of piriform inhibition. Permanent lesions that alter the progression of amygdala or hippocampal kindling have included the destruction of the central piriform cortex with ibotenate (198, 199), electrical ablation of the ipsilateral piriform, and knife-cut disconnection of the anterior piriform (200). These increased the number of stimulations to achieve kindling, prolonging either during the early or later phases, and increased the post-kindling seizure threshold. Other approaches such as injecting the anterior piriform, or bilateral radio-ablation of the area tempestas did not alter kindling (91), re-enforcing that it is the posterior piriform, which is the critical site for discharge propagation.

Chemical modulation of the piriform cortex also can alter the course of amygdala kindling. Microinjection of a GABA_A_ receptor agonist, or an NMDA receptor antagonist reduces the duration of kindled afterdischarges (92, 201). Microinjection of vigabatrin, an antiepileptic medication, which elevates local GABA levels, inhibited seizures in previously kindled animals, showing greatest effect when applied to the central piriform cortex (201, 202). Finally, local application of adenosine to the piriform cortex (an endogenous neurotransmitter that may have an antiepileptic effect by decreasing glutamate release), inhibited kindling from both the amygdala and the hippocampus (203, 204). Kindling of the amygdala can also be blocked by lesions at the dorsal claustrum (205), demonstrating that the piriform cortex is not the only critical structure in limbic epileptogenesis.

In summary, the available evidence shows that either chemical or repeated electrical stimulation applied to limbic sites can produce complex changes in the piriform cortex, which ultimately results in increased piriform cortex excitability. Therefore, the piriform cortex can provide a pathway for focal epileptogenesis, via the facilitation and widespread distribution of epileptic discharges.

As a corollary, we should consider whether the piriform cortex has any influence on the progression to intractable epilepsy. Defined in clinical populations as ongoing seizures despite adequate trials of two appropriate and tolerated medications (206), medication resistance in epilepsy is likely to be a multifactorial process (207), and is often related to the intrinsic severity of the epilepsy syndrome (208). The epilepsy most strongly associated with piriform cortex involvement is temporal lobe epilepsy (as discussed in Sections “Human Seizures with Olfactory Auras Tell us About Epileptic Involvement of the Olfactory Network” and “The Impact of Epilepsy on Olfaction and its Imaging Correlates in Human Beings”), which has high rates of medical intractability that may either be present from the onset, or develop over time (209). Hippocampal atrophy is a particular marker for progression to intractability in this group, and an association between hippocampal atrophy and piriform atrophy has been noted (10). High initial seizure frequency and the occurrence of status epilepticus are known to cause piriform cortex injury and are also risk factors for intractability (210). A further mechanism of pharmacoresistance is the expression of the multi-drug transporter P-glycoprotein, which can cause efflux of medications from epileptogenic sites (211). Marked P-glycoprotein expression has been seen at both hippocampus and piriform cortex in phenobarbitone-resistant rat models (212). However, there may be significant inter-species variation for this mechanism, and recent *in vivo* human imaging of P-glycoprotein did not detect significant changes at the piriform cortex (213). Lastly, alterations in neural networks due to axonal sprouting and synaptic reorganization may contribute to pharmacoresistance (214). Therefore, the piriform cortex has anatomical and functional characteristics that position it to contribute to the phenomena associated with intractability.

## The Impact of Epilepsy on Olfaction and Its Imaging Correlates in Human Beings

### Olfactory function is impaired by focal epilepsy

A common theme in focal epilepsy is that overlap of epileptic regions with sensory networks produces dysfunction of that modality (215). In patients with temporal lobe epilepsy, many aspects of olfactory function are abnormal (115), which is most likely caused by epileptic involvement of the olfactory network.

The threshold for detection of odors is normal for people with temporal lobe epilepsy, on standard testing with n-butanol or phenyl ethyl alcohol (124, 216–220). However, some studies have found reduced sensitivity for odors by using broader panels of odorants (115, 221). The occurrence of seizures may transiently alter odor detection thresholds, with heightened olfactory sensitivity during the seizure prodrome, and reduced sensitivity lasting for hours or days in the post-ictal phase (115).

In contrast, odor discrimination, memory, and identification/naming are all commonly impaired in temporal lobe epilepsy. Odor discrimination relies on the piriform cortex, orbitofrontal regions, and the hippocampus (222), and failure on this task reflects dysfunction of these networks (219, 220, 223, 224), although this deficit has not been confirmed on all studies (217). Single-nostril presentation of odorants lateralizes the deficit to the same side as the epileptic focus (220).

Memory recall of odors activates an extensive network including olfactory cortex, semantic networks, and attention systems (13). Impaired odor memory has been demonstrated with a variety of protocols (216, 220). Some studies have detected abnormality only in left sided (225), or in right-sided temporal lobe epilepsy (226), probably related to the relative involvement of the autobiographical memory network versus semantic networks on a given task (227). Single-nostril presentation again shows an ipsilateral deficit, being more pronounced in left sided epilepsy (223).

Identification of odors, for example by selecting from a list of names, is also impaired (124, 217, 219). This deficit occurs equally with left and right-sided temporal lobe epilepsy (216, 225) or can have a right temporal lobe predominance (218). Correct odor identification activates olfactory, limbic and semantic networks, plus other primary cortical areas (68), but may have more pronounced involvement of the non-dominant hemisphere when non-verbal identification is used (228, 229).

Olfactory function in patients with generalized or extra-temporal focal epilepsy has rarely been tested. Impaired odor identification was found in a mixed group mostly with generalized epilepsy (225). Another group with extra-temporal epilepsies had normal odor detection, discrimination, and memory (220). This may be surprising in light of the EEG-fMRI findings indicating common involvement of the piriform cortex in some extra-temporal focal epilepsies, although more behavioral data is clearly needed to address this discordance.

### Neuroimaging of piriform cortex shows olfactory dysfunction in focal epilepsy

Multiple neuroimaging modalities have shown changes to piriform cortex in focal epilepsy, which parallel the dysfunction of olfactory processing we have described above.

Volumetric MRI shows piriform cortex atrophy in temporal lobe epilepsy. This was examined by manual tracing of the temporal and periamygdaloid cortex, identifying reduced volume on the same side as the epileptic focus (10). This effect is greater with right-sided epilepsy. Piriform cortex atrophy is bilateral in a subgroup of patients with left temporal lobe epilepsy. There is a significant correlation between atrophy of piriform cortex and atrophy of the hippocampus, amygdala, and entorhinal cortex, indicating that the piriform changes are not isolated, but are part of a distributed network effect. The olfactory bulb volume is also reduced in temporal lobe epilepsy (230), which may be a “top-down” effect driven by pathology within primary olfactory cortex.

In frontal lobe epilepsy, voxel-based morphometry has surprisingly shown increased volumes of the piriform cortex and amygdala bilaterally compared to controls, and no regions of atrophy were found (231). The meaning of increased gray matter volume in this context is uncertain.

Chemosensory evoked potentials (CSERPs) can tell us about the relative timing of olfactory processing. In temporal lobe epilepsy, evoked potentials are delayed when an odor is presented to the side of the epileptic focus (232). This effect was most pronounced in right temporal lobe epilepsy, reflecting the relative importance of the right hemisphere in olfaction.

The functional activity of olfactory brain regions in epilepsy has been investigated with positron emission tomography (PET) using an [^15^O]-H_2_O tracer (233). People with temporal lobe epilepsy failed to activate the ipsilateral piriform cortex, amygdala, and anterior insula when smelling various odors. Furthermore, when smelling familiar (nameable) odors patients with left mesial temporal lobe epilepsy failed to activate left inferior frontal cortex, which the authors suggest may be due to impairment of connections between olfactory and semantic networks.

PET using a [^11^C]-flumazenil tracer has been used to probe GABA_A_ receptor expression. In a group of patients with focal epilepsy from all lobes, flumazenil binding was inversely correlated with seizure frequency in the frontal piriform cortex (151). The finding that people with more seizures have the weaker expression of GABA_A_ receptors, suggests that altered GABAergic inhibition in the piriform cortex may be a consequence of increased seizure frequency, and potentially even a cause for frequent seizures.

## Can Stimulation of the Piriform Cortex be Used Therapeutically?

### Aborting seizures with an olfactory stimulus

As early as 1881, Gowers suggested that the application of a strong aroma, such as ammonia or amyl nitrite, may in some cases arrest the course of a seizure (234). Other historical accounts have described the use of other unpleasant odors such as “shoe-smell” (235). Setting aside any direct pharmacological effect of these odors, a plausible hypothesis is that strong physiological activation of olfactory cortex can temporarily prevent or disrupt the progression of epileptic discharges. An alternative interpretation would be that the smell produces a change in cognitive state, for example alertness, which is less permissive for seizures to evolve.

In a detailed clinical account of this technique, Efron describes a woman with “uncinate” seizures, who had an exceptionally long olfactory prodrome that would reliably evolve into an olfactory hallucination and eventually a generalized convulsion (236, 237). Medial temporal epileptic discharges during her attacks were confirmed using sphenoidal electrodes. Smelling an unpleasant odor in the early phase of her attacks (such as hydrogen sulfide, dimercaprol or jasmine) would reliably prevent the seizure progressing, and she was able to use this approach for seizure control.

Experimental evidence from the amygdala-kindling model supports olfactory stimulation as a plausible treatment. After rats had been fully kindled, olfactory presentation of toluene was effective at preventing seizures (238). Smelling either toluene or ammonia increased the amygdala stimulation threshold for inducing events, and with ammonia the seizure duration was also decreased.

Conversely, there are rare reports of seizures being triggered by an olfactory stimulus. During depth-electrode recording from the amygdala in an awake patient (239), smelling various odors induced an amygdala discharge accompanied by similar symptoms to her usual focal seizures.

The therapeutic use of an odor to abort seizures is unfortunately only applicable to a very small number of patients. It requires that the patient have a long aura phase with preserved awareness where they can take this action, and the even then, the probability of this intervention being successful is unknown. Nonetheless, it does demonstrate an important mechanism of relevance to more invasive treatment approaches.

### Deep brain stimulation of piriform cortex

If physiological stimulation of piriform cortex can interrupt or prevent seizures, then perhaps direct electrical stimulation at this site could have the same effect. Deep brain stimulation (DBS) for focal epilepsy in human beings has shown promising results, particularly with stimulation of the anterior nucleus of the thalamus (240, 241). However, piriform DBS has not been performed in human beings, and only a few studies have been done in experimental animals.

In rats, low-frequency electrical stimulation of the piriform cortex at 1Hz has been used. With an amygdala-kindling model, piriform stimulation inhibits the kindling process (242), and also decreases the incidence of generalized seizures in fully kindled animals. More specifically, this was achieved by stimulation of the ipsilateral central piriform cortex, with contralateral stimulation being less effective (243, 244). On the other hand, when the kindling was initially directed at the piriform cortex, inhibitory piriform stimulation was not effective (245). Therefore, piriform stimulation may be most useful when it is a secondary relay for discharges, rather than the primary epileptogenic site, with the aim being to preventing piriform-mediated amplification and distribution of widespread discharges.

Therefore, whether piriform cortex or endopiriform nucleus DBS may be of any benefit in human focal epilepsy is currently unknown. At a minimum, further studies of DBS to these targets in animal models of epilepsy will be needed to approach this question.

## Case Report: Surgical Treatment of Possible Piriform Epilepsy

Surgical intervention involving the piriform cortex may be beneficial for carefully selected patients, but poses a particular diagnostic and anatomical challenge. Here, we report a 37-year-old woman who had seizures from the second year of life, which consisted of an aura of feeling scared, followed by screaming and wild flailing of all limbs or cycling leg movements. She did not have an olfactory aura. Events were brief, lasting less than 1 min. Ictal scalp EEG showed bitemporal rhythmic delta. High-resolution MRI did not identify any lesion. FDG-PET was non-localizing. One ictal-interictal SPECT suggested right orbitofrontal hyperperfusion.

**Figure 4:**
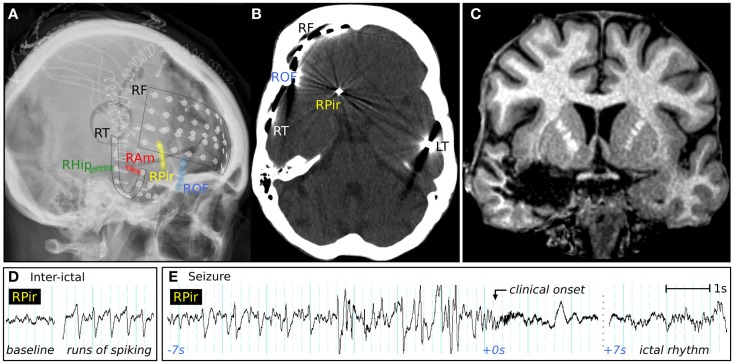
**Clinical imaging of a patient with possible piriform epilepsy**. **(A)** Lateral skull X-ray showing positions of intracranial electrodes. RF, right frontal subdural electrodes; RT, right temporal subdural electrode strip; RHip, right hippocampal depth electrode; RAm, right amygdala depth electrodes; RPir, right piriform electrodes; ROF, right orbitofrontal subdural electrodes; LT, left temporal subdural electrode strip. **(B)** CT performed in the piriform axis showing the position of the most inferomesial RPir electrode contact, in orbitofrontal cortex adjacent to frontal piriform cortex. **(C)** Coronal T1-weighted MRI, showing posterior extent of surgical resection, with removal of right frontal piriform cortex. **(D)** EEG recorded from most inferomesial RPir electrode, showing trains of inter-ictal spiking, and **(E)** a seizure from sleep, with progressively building discharges, then gamma activity and attenuation, followed 7 s later by an evolving ictal rhythm. At the “clinical onset,” there was explosive onset of screaming and flailing movements of the limbs.

Video-EEG monitoring was performed with multiple frontal and temporal intracranial electrodes, including a depth-electrode targeting the frontal piriform cortex, placed via the lateral frontal lobe (Figures 4A,B). Inter-ictal recordings showed bursts of epileptiform gamma over the orbitofrontal cortex, and spiking at the hippocampus and temporal pole. Sub-clinical electrographic seizures (Figure 4D) were recorded from the piriform cortex electrode, showing 1–2 min runs of rhythmic sharp waves. Her stereotyped clinical seizures (Figure 4E) showed attenuation and gamma frequencies at the piriform and orbitofrontal electrodes, then an evolving ictal rhythm at these locations and over the right temporal lobe.

A right temporal lobectomy was performed, and was extended into the frontal lobe to remove frontal piriform cortex, along with posterior parts of the inferior frontal gyrus and lateral orbital gyrus. Resection was also extended to remove temporal piriform cortex, the antero-inferior amygdala, and the hippocampus (Figure 4C). Histology of the orbitofrontal tissue showed some disorganized architecture and prominent single white matter neurons, interpreted by the neuropathologist as possible focal cortical dysplasia (MCD 1) although no balloon cells or dysmorphic neurons were seen. No tissue abnormality was found in temporal lobe structures.

Following surgery, she had a marked reduction of seizure frequency, from several events per day to occasional and mainly nocturnal events. There was immediate improvement in her responsiveness and speed of processing compared to her preoperative psychomotor slowness. Although the semiology in this case simultaneously suggested frontal lobe (ictal hypermotor activity) and amygdala activity (prolonged episodes of fear), the implantation identified orbitofrontal cortex or frontal piriform cortex as the most likely regions of onset. Resection of these structures was by necessity incomplete, in part because of the dangerous proximity of the middle cerebral artery and other vessels traversing the anterior perforated substance.

## Discussion

In this review, we have examined several lines of evidence that associate the piriform cortex with focal epilepsy. The central question is therefore, what role does the piriform cortex play?

It is clearly the generator of seizures in animal models where chemical or electrical stimulation is applied directly to the piriform cortex. The human piriform cortex is very likely to share this exquisite sensitivity to pro-convulsive stimulation. However, only very rare cases of human epilepsy arising directly from piriform cortex have been described, such as that of Mizobuchi et al. (118), and arguably the case report described above.

Conversely, the piriform cortex will be an unrelated bystander in some forms of epilepsy, with no role in seizure onset or spread. Focal seizures from the occipital or parietal lobes may be examples in this category, although only limited data about the piriform cortex has been reported for these patients so far (154, 220, 225).

Of greater clinical relevance, the piriform cortex is a common target of discharge spread, particularly in frontal lobe and temporal lobe epilepsy. This is indicated by the site of lesions that can produce an olfactory epileptic aura (102), the impact of fronto-temporal epilepsy on olfactory function (115), and the detection of piriform cortex activity on EEG-fMRI in these cases (151).

A role for the piriform cortex during human epileptogenesis is probable, but remains to be confirmed. Its tendency to suffer preferential neuronal loss following seizures, as observed in both human status epilepticus (156), and in animal models of induced epilepsy (181), may lead to electrophysiological and local microcircuit changes (185), which result in piriform hyper-excitability. We hypothesize that when the piriform cortex is a target of discharge spread, it can be readily recruited as a secondary hyper-excitable node in the epileptic network by this mechanism. However, inhibition of the piriform cortex only partially blocks the development of epilepsy (86), meaning that it is still possible for epileptogenesis to occur via other less sensitive pathways.

Subsequently the piriform cortex can act as a distributor of epileptic discharges, by facilitating seizures with a limbic origin to spread into olfactory and cortical networks, and vice versa. The evidence for this comes from the amygdala-kindling model of focal epilepsy (3), and clinical descriptions of aura progression (116).

This predisposition of piriform cortex to become involved in focal epilepsy may be understood from the perspective of the architecture that has developed to achieve its normal function. The high inter-connectivity of excitatory neurons provides the basis for a spatially distributed representation of odors, with an intrinsic method for template completion and pattern matching (37). However, this same architecture makes it prone to forming hyper-excitable local networks if local inhibitory circuits are altered or lost. Furthermore, strong reciprocal connectivity to nearby structures such as the olfactory bulb, amygdala, and hippocampus-entorhinal cortex are essential for top-down modulation of olfactory inputs, olfactory memory, and the processing of emotional salience. However, these loops pose the risk of becoming reentrant circuits that sustain seizure activity (3).

Therefore the piriform cortex is highly relevant to the understanding of human focal epilepsy arising from the temporal or frontal lobes. It is a common node of discharge spread, can be injured and kindled by seizure activity, and may be involved in the facilitation and distribution of epileptic discharges throughout limbic and cortical networks. It is a potential target for invasive therapies, including EEG recording and surgical resection, and its unique properties and anatomical relationships must be taken into account.

## Conflict of Interest Statement

The authors declare that the research was conducted in the absence of any commercial or financial relationships that could be construed as a potential conflict of interest.
